# The effectiveness of high intensity laser therapy in the patients with lumbar disc herniation: A protocol of randomized placebo-controlled trial

**DOI:** 10.1097/MD.0000000000022520

**Published:** 2020-10-09

**Authors:** Youyi Huang, Daxin Gao

**Affiliations:** a School of Medicine, Nanchang University, Jiangxi; b Department of Orthopedics, West China-Guangan Hospital, Sichuan, China.

**Keywords:** high-intensity laser therapy, Lumbar disc herniation, pain control, protocol., random

## Abstract

**Background:**

There is no consensus in existing literature on the pulse power, application time, frequency and the dose of energy of laser therapy for the patients. Therefore, we conducted this research for the assessment of safety and efficiency of ultrasound and high-intensity laser therapy (HILT) in the lumbar disc herniation (LDH) patients.

**Methods:**

Our present research was approved by the institutional review board in the West China-Guangan Hospital. All the participants would acquire the written informed consent. From December 2020 to December 2021, we will conduct a prospective evaluation via a senior surgeon for 1 hundred LDH patients who plan to undergo the conservative treatment at our hospital. In this research, the inclusion criteria contained: the patients with lumbar disc herniation diagnosed by lumbar MRI; the patients with no history of trauma or congenital abnormalities; and the patients with sufficient psychological ability to understand and then answer the questions raised in assessment scale. The participants were randomly divided into the control group or HILT group after performing the examination of baseline. The main outcome was the pain score of visual analog scale. The other results contained the adverse effects, back range of motion as well as functional scores.

**Conclusions:**

We assumed that the HILT is as effective as the ultrasound therapy in treating pain for LDH.

**Trial registration:**

This study protocol was registered in Research Registry (researchregistry5975).

## 1 Introduction

About 5% to 15% of the low back pain patients suffer from the lumbar disc herniation (LDH).^[1–3]^ LDH is a kind of limited disc material displacement beyond the edge of normal intervertebral disc space, which is most familiar cause of the sciatica and affects 1 to 5 percent of population every year.^[4]^ The treatment of LDH is of great significance in managing pain, developing the disability and chronic pain and preventing the recurrence, and then accelerating the process of returning to work. The first-line management for the LDH is non-surgical treatment, which may include the pharmacologic and physical therapies, and/or the injection of epidural steroid.^[5–9]^

The high-intensity laser therapy (HILT), which involves laser radiation with high-intensity, is a kind of novel, powerful and painless mode and has significant effects on relieving pain. The HILT possesses its own photomechanical, photothermal, and the photo-chemical properties, and it has many therapeutic effects, containing anti-edema, analgesic and the biological stimulation.^[10–15]^ Another benefit of HILT, in particular the neodymium-doped yttrium aluminum garnet laser, is its higher power and penetration depth to the deep tissues.^[16]^ The application of HILT obviously decreases the degrees of pain in the chronic and acute diseases, for instance, carpal tunnel syndrome, chronic osteoarthritis, and rheumatoid arthritis, shoulder pain, the injuries of knee, fibromyalgia, as well as the pain after operation.^[11,17–20]^

There is no consensus in existing literature on the pulse power, application time, frequency and the dose of energy of laser therapy for the patients. At present, there are a few investigations on the effects of HILT treatment on the lateral epicondylitis, frozen shoulder, and cervical radiculopathy, knee arthritis, LDH as well as post-mastectomy. Nevertheless, in the chronic lumbar radiculopathy patients, the literature on the treatment of HILT is limited. Therefore, we conducted this research for the assessment of safety and efficiency of ultrasound and HILT in the LDH patients. We assumed that the HILT is as effective as the ultrasound therapy in treating pain for LDH.

## 2 Material and method

### 2.1 Study design

Our present research was approved by the institutional review board in the West China-Guangan Hospital (GA20201703). All the participants would acquire the written informed consent. From December 2020 to December 2021, we will conduct a prospective evaluation via a senior surgeon for one hundred LDH patients who plan to undergo the conservative treatment at our hospital. Our present investigation was registered with the Research Registry (researchregistry5975) before registration started. All the surgeons, patients, statisticians, as well as research assistants who participated in this study were not aware of the group assignment.

### 2.2 Patient enrollment

In this research, the inclusion criteria contained: the patients with lumbar disc herniation diagnosed by lumbar MRI; the patients with no history of trauma or congenital abnormalities; and the patients with sufficient psychological ability to understand and then answer the questions raised in assessment scale. Patients with the history of severe osteoporosis or lumbar injections in past 4 weeks, and the patients with inflammatory pain, acute trauma, lumbar surgery, lumbar instability and neurological disorders, patients with severe diseases and severe or uncontrolled cardiovascular, as well as the patients undergoing physical treatment in the past 3 months will be excluded.

### 2.3 Randomization

**Figure 1:**
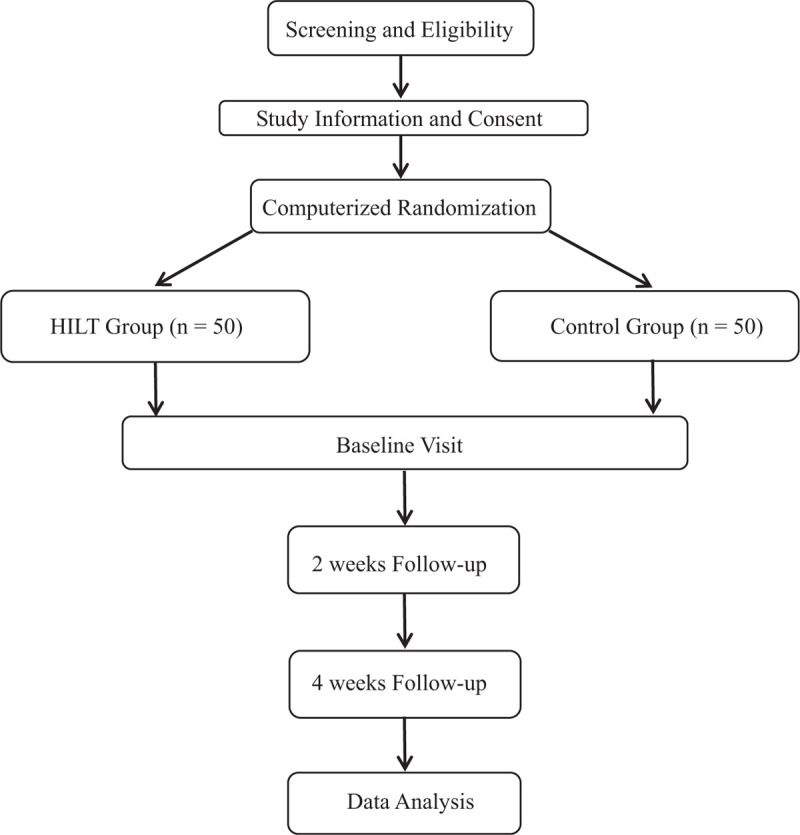
Consolidated standards of reporting trials statement flow diagram.

The participants were randomly divided into the control group or HILT group after performing the examination of baseline. The computer random number generator was applied for the random grouping. It priori produced one hundred random integers, and the random number already exist before this research starts. The randomly assigned, sequentially numbered and separated index cards were prepared. These above index cards were folded, and then kept in the opaque sealed envelopes. A researcher who did not know the baseline examination opened these envelopes and determined the interventions in accordance with the assignments of group (Fig. 1).

### 2.4 Interventions and control

The intervention group received the HILT (BTL 6000) after twenty minutes of hot compress for 2 weeks, and 5 days a week, in all, the intervention group received 10 times high intensity laser treatment. The device was set at 10 W power, 25 Hz frequency and 12 j/cm^2^ dose, the lumbar area exceeding 25 cm^2^ was stimulated with a biological stimulation mode for 4 minutes, and then performing the continuous pattern for 6 minutes with the dosage of 120 j/cm^2^ and 7 W power. The control group utilized the mobile ultrasound equipment during the treatment. The ultrasound was utilized for ten minutes at a power of 1.5 watt/cm in lumbar paravertebral region. Conductivity gel could be applied to enhance the efficiency of absorption and accomplish the thermal effects of deep muscle.

### 2.5 Outcomes

The main outcome was the pain score of visual analog scale (VAS). VAS has been proved as an effective and reliable method for the pain measurement. On a 10-centimeter line, the score of 0 represented no pain, and 10 represented maximum pain. On this 10-centimeter line, the patients needed to show the severity of their low back pain. Afterwards, using a ruler to measure the distance from 0 to the marked point.

The other results contained the adverse effects, back range of motion (ROM), as well as functional scores [Oswestry Disability Index, Roland Disability Questionnaire]. Roland Disability Questionnaire is a kind of sensitive, effective and reliable questionnaire, which could be used for the measurement of the functional level of patients in conducing daily tasks. The score ranges are between 0 and 24, and the patient is asked to put a marker next to each proper statement in the 24 statements list. Oswestry disability index consists of ten questions that evaluate weight lifting, personal care, pain, standing and sitting, walking, and sleeping, social life, the pain variation level and trave, with each question scoring from 0 point to 5 point. The highest score is 50. Multiply the total score by 2 to obtain the percentage result. For ease of use, a ROM goniometer was applied for the measurement of the spine ROM: the extension and flexion of the spine was measured by sagittal plane meter, and lateral flexion was measured via lateral plane meter. The measurements of ROM were implemented twice via 2 independent observers prior to treatment initiation and during a 4-week follow-up examination.

### 2.6 Sample size determination

The power and sample size were calculated through using the statistical software of nQuery Advisor (version 6.0). The VAS difference between the 2 repeated measurements can reach 80% with 0.05 level of alpha, 1.5 standard deviation, and when the correlation between observed values of the same participant was 0.7 and the sample size of the intervention group and the control group is 50 and 50 respectively.

### 2.7 Statistical analysis

The software of SPSS v22.0 could be utilized to conduct the statistical analyses (IBM, Chicago, IL). KolmogoroveSmirnov test was applied to test the consistency between the normal distribution and the data. The continuous variables were compared with the independent 2 samples *t*-test, and for the categorical variables comparison, it can be implemented with the pearson Chi Square test. The obtained results were assessed in the 95% confidence interval with *P* < .05 significance level.

## 3 Discussion

LDH is 1 of the most familiar causes of the low back pain. According to a survey, about 70% to 85% of people have the low back pain, with an annual incidence of 15% to 45% and an annual medical cost of 26.3 million dollars for the LDH, which creates a serious financial burden on the society. Thus, the focus of research on the disease is to find a cost-effective treatment approach. Laser therapy is a kind of painless, non-invasive and it can be easily managed in a wide variety of conditions in the primary care setting. We conducted this research for the assessment of safety and efficiency of ultrasound and HILT in the LDH patients. We assumed that the HILT is as effective as the ultrasound therapy in treating pain for LDH.

## Author contributions

**Conceptualization:** Youyi Huang.

**Data curation:** Youyi Huang, Daxin Gao.

**Formal analysis:** Youyi Huang, Daxin Gao.

**Funding acquisition:** Daxin Gao.

**Investigation:** Daxin Gao.

**Methodology:** Youyi Huang.

**Resources:** Daxin Gao.

**Software:** Daxin Gao.

**Supervision:** Daxin Gao.

**Validation:** Youyi Huang.

**Visualization:** Youyi Huang.

**Writing – original draft:** Youyi Huang.

**Writing – review and editing:** Daxin Gao.

## Abbreviations

Abbreviations: HILT = high-intensity laser therapy, LDH = lumbar disc herniation, VAS = visual analog scale, ROM = range of motion.
